# The sodium glucose cotransporter 2 inhibitor empagliflozin does not prolong QT interval in a thorough QT (TQT) study

**DOI:** 10.1186/1475-2840-12-70

**Published:** 2013-04-24

**Authors:** Arne Ring, Tobias Brand, Sreeraj Macha, Kerstin Breithaupt-Groegler, Gudrun Simons, Beate Walter, Hans J Woerle, Uli C Broedl

**Affiliations:** 1 Boehringer Ingelheim Pharma GmbH & Co. KG, Birkendorfer Str. 65, Biberach an der Riss, 88397, Germany; 2 Institute for Epidemiology and Medical Biometry, University Ulm, Schwabstr. 13, Ulm, 8975, Germany; 3 Boehringer Ingelheim Pharmaceuticals Inc., Schwabstr. 13, Ridgefield, CT, 06877-0368, Germany; 4 kbr- Clinical Pharmacology Services, Frankfurt, Germany; 5 Independent Consultant, Limburgerhof, Germany; 6 Boehringer Ingelheim Pharma GmbH & Co. KG, Binger Str. 173, Ingelheim, 55216, Germany

**Keywords:** Empagliflozin, SGLT2 inhibitor, Diabetes, QT interval, ECG

## Abstract

**Background:**

Empagliflozin is a potent, selective sodium glucose cotransporter 2 (SGLT2) inhibitor in development as an oral antidiabetic treatment. This QT interval study assessed potential effects of empagliflozin on ventricular repolarisation and other electrocardiogram (ECG) parameters.

**Methods:**

A randomised, placebo-controlled, single-dose, double-blind, five-period crossover study incorporating a novel double-placebo period design to reduce sample size, while maintaining full statistical power. Treatments: single empagliflozin doses of 25 mg (therapeutic) and 200 mg (supratherapeutic), matching placebo and open-label moxifloxacin 400 mg (positive control). Triplicate 12-lead ECGs of 10 second duration were recorded at baseline and during the first 24 hours after dosing. The primary endpoint was mean change from baseline (MCfB) in the population heart rate-corrected QT interval (QTcN) between 1–4 hours after dosing.

**Results:**

Thirty volunteers (16 male, 14 female, mean [range] age: 34.5 [18–52] years) were randomised. The placebo-corrected MCfB in QTcN 1–4 hours after dosing was 0.6 (90% CI: -0.7, 1.9) ms and -0.2 (-1.4, 0.9) ms for empagliflozin 25 mg and 200 mg, respectively, below the ICH E14 defined threshold of regulatory concern 10 ms. Assay sensitivity was confirmed by a placebo-corrected MCfB in QTcN 2–4 hours post-dose of 12.4 (10.7, 14.1) ms with moxifloxacin 400 mg. Empagliflozin tolerability was good for all volunteers; 23.3% experienced adverse events (AEs) with empagliflozin and 27.6% with placebo. The most frequent AE was nasopharyngitis.

**Conclusions/interpretation:**

Single doses of empagliflozin 25 mg and 200 mg were not associated with QTcN prolongation and were well tolerated in healthy volunteers.

**Trial registration:**

ClinicalTrials.gov: NCT01195675

## Background

The sodium glucose cotransporter 2 (SGLT2) plays an important role in glucose homeostasis, being responsible for around 90% of renal glucose reabsorption [1], and its inhibition represents a novel approach for the treatment of type 2 diabetes mellitus (T2DM). SGLT2 inhibitors act by blocking renal glucose reabsorption via an insulin-independent mode of action in order to eliminate excess glucose from the body via the urine [1,2]. Besides their proven efficacy in lowering plasma glucose levels, these agents have also been shown to have potential benefits for improving other cardiovascular risk factors, such as body weight and blood pressure, while being well tolerated [3]–[7].

Empagliflozin is a potent and highly selective SGLT2 inhibitor that has been shown to reduce plasma glucose levels in patients with T2DM with a low risk of hypoglycaemia [6,8,9]. Single doses of empagliflozin (≥800 mg) were rapidly absorbed, reaching peak levels after 1.5–2.5 hours, with a terminal elimination half-life of around 10–13 hours for doses ≥10 mg, indicating that the drug is suitable for once-daily administration. In addition, no clinically relevant effects of food on drug exposure were reported [10]. No evidence of cardiac safety issues have been observed in pre-clinical or Phase I studies. No relevant interactions with the human ether-a-go-go related gene (hERG)-mediated potassium current were measured in transfected human embryonic kidney cells (HEK293 cells), and empagliflozin doses of ≥10 μM had no effect on action potential configuration or contractile function of guinea pig papillary muscle (unpublished data).

QT interval prolongation can be associated with life-threatening arrhythmias [11,12], and has been documented with a number of drugs [13]. It is important to establish the cardiovascular safety of new antidiabetic drugs as patients with T2DM have a higher risk of cardiovascular disease (CVD) and CVD-related mortality [14,15] that can be confounded by hypoglycaemia and other glucose-independent treatment effects [16,17]. CVD is still the major cause of death in patients with T2DM [14,18], despite glycaemic control measures designed to reduce vascular complications related to glucotoxicity [19,20].

The aim of this study was to confirm the absence of QT effects with therapeutic and supratherapeutic doses of empagliflozin compared with moxifloxacin as a positive control and placebo, using a new five-period crossover study design, utilising two placebo periods instead of one.

## Methods

### Study design

This thorough QT study (TQT) was a randomised, double-blind (moxifloxacin open), placebo-controlled crossover trial which included the following treatments: 25 mg and 200 mg empagliflozin (Boehringer Ingelheim Pharma GmbH & Co. KG); 400 mg open-label moxifloxacin (Avalox®; Bayer Vital, Leverkusen, Germany); and two placebo periods, with a washout of at least seven days between treatments. Trial medication was administered in the morning after an overnight fast.

Deviating from the conventional four-period design for TQT studies, this study utilised a five-period design with two placebo periods [21,22]. Although this design has not been implemented in a study before, its advantages have been previously recognised by other authors [21,23]. The design is considered to be advantageous for TQT trials because all of the required between-treatment comparisons of ECG intervals are comparisons of active drug with placebo. Doubling the number of placebo periods allows the overall sample size to be reduced [21,23] (Table 1; see sample size section below).

**Table 1:** Comparison of four- and five-period crossover designs for TQT studies leading to the same statistical power

	**Typical four-period** **TQT design**	**New five-period** **TQT design**
Placebo periods	1	2
Placebo sessions	40	60
Supratherapeutic dose sessions	40	30
Therapeutic dose sessions	40	30
Active control sessions	40	30
Total number of sessions	160	150

TQT, thorough QT.

The single oral dose of 25 mg empagliflozin is the expected daily therapeutic dose, based on an expected mean maximum plasma concentration (C_max_) of 505 nmol/L and a mean time to maximum plasma concentration (t_max_) of 2.2 hours in healthy volunteers [10]. The single oral dose of 200 mg empagliflozin was chosen as the supratherapeutic dose, based on an expected mean C_max_ of 3490 nmol/L (~7-fold higher than the therapeutic dose) and a mean t_max_ of 1.8 hours in healthy volunteers [10].

Moxifloxacin 400 mg was used as a positive control according to the ICH E14 guidance [24], as this treatment was shown to prolong the heart rate-corrected QT interval by approximately 6–15 ms compared to placebo in several clinical trials in healthy volunteers [25]–[28].

The trial was conducted in accordance with the Declaration of Helsinki and the International Conference of Harmonisation Tripartite Guideline for Good Clinical Practice. The study was approved by the local Ethical Committee (*Landesärztekammer Baden-Württemberg*, Stuttgart, Germany) and the German Competent Authority (*Bundesinstitut für Arzneimittel und Medizinprodukte*, Bonn, Germany). All participants provided written, informed consent prior to the start of the study.

### Participants

Healthy male and female volunteers, aged between ≥18 and ≤55 years with body mass index (BMI) ≥18.5 kg/m^2^ and ≤29.9 kg/m^2^, who were judged to be in good health based on medical history, physical examination, ECG and routine laboratory evaluations were eligible to enter the trial. Participants were excluded if there was evidence of a clinically relevant disease, history of risk factors for QT prolongation (e.g. heart failure, hypokalaemia, family history of Long QT Syndrome), any clinically relevant deviations in blood pressure, pulse or ECG, or a marked baseline prolongation of QT/QTc interval (e.g. repeated occurrence of >450 ms interval). No concomitant therapy was allowed except for oral contraceptives. Volunteers were recruited at a single centre: Human Pharmacology Centre, Boehringer Ingelheim Pharma GmbH & Co. KG, Biberach, Germany.

### Assessments

ECG recordings were made at screening and at the end of the study as part of the safety evaluation. For the QTc evaluation, ECG recordings were made on study drug administration days (as ECG profiles) at 60, 50 and 40 minutes pre-dose (to derive the period-specific baseline), and at 0.5, 1, 1.5, 2, 2.5, 3, 4, 6, 8, 12 and 24 hours post-dose (last three time points omitted for moxifloxacin treatment). At each time point, triplicate standard 12-lead ECGs (I, II, III, aVR, aVL, aVF, V1–V6) of 10 seconds’ duration each were recorded after at least 5 minutes’ rest in the supine position using CardioSoft ECG recording machines (GE Healthcare, Freiburg, Germany). ECGs were sent to a central laboratory for evaluation of PR, QRS, RR and QT intervals. The semi-automatic approach used for determination of the fiducial points provided an automatic pre-assessment using a computer algorithm, which was reviewed by a specialist and adjusted if necessary. This review was performed blinded with regard to treatment and time point.

Data from four cardiac cycles per ECG were averaged. A population heart rate-corrected QT interval (QTcN) was derived by determining the exponent δ of the relationship: QT ~ (1000 / RR)^δ^ (where δ is the regression effect of the covariate) using a linear mixed model on log-transformed RR and QT data (measured in ms). Data from triplicate ECGs were then averaged for each time point. Correction for heart rate is necessary to allow QT interval comparisons to be independent of potential changes in heart rate (e.g. due to natural variability or circadian rhythm), and this method has been shown to be superior than others commonly used for this purpose [29].

Blood samples for pharmacokinetic measurements were taken one hour before drug administration and at the same post-dose time points as ECG measurements, except for the moxifloxacin periods. Empagliflozin concentrations in plasma were determined using a validated high performance liquid chromatography, tandem mass spectrometry (HPLC-MS/MS) assay with a lower limit of quantification of 1.11 nmol/L (0.5 ng/mL). Results were calculated using peak area ratios and calibration curves were created using weighted (1/x2) quadratic regression. This method demonstrated acceptable precision and accuracy of quality control samples, and the stability of empagliflozin and [^13^C_6_]-empagliflozin was verified under a variety of conditions. Pharmacokinetic parameters included C_max_, t_max_ which were determined directly from the plasma concentration-time profiles of each, and area under the plasma concentration curve from zero to the last quantifiable time point (AUC_0–tz_), calculated using the linear trapezoidal method for ascending concentrations and the log trapezoidal method for descending concentrations. Pharmacokinetic parameters were determined using WinNonlin™ software v5.2 (Pharsight Corporation, Mountain View, California, USA).

### Endpoints

The primary endpoint was the mean change from baseline (MCfB) in QTcN which was the mean QTcN derived from ECGs obtained 1–4 hours post-dose minus mean QTcN from baseline ECGs obtained pre-dose at each visit. Plasma concentrations of both empagliflozin and moxifloxacin were expected to reach peak levels within this three-hour window [10,25], ensuring the most relevant levels of drug exposure. Use of pre-dose period baseline ECGs has been shown to be the least variable method of baseline correction [30], and three triplicate ECG recordings were used in order to reduce the baseline variance further [31].

Secondary endpoints were the changes from period baseline in QTcN at any time point between 0.5–24 hours after dosing. In addition, the MCfB 2–4 hours after dosing (for assessment of the moxifloxacin effect) [32], and the MCfB of all ECGs taken 0.5–24 hours after dosing were evaluated. Safety and tolerability were evaluated based on physical examination, vital signs, ECG, clinical laboratory tests, adverse events (AEs) and the physician’s assessment of global tolerability.

### Sample size

Detailed considerations relating to the sample size calculation have been published previously [22]. In summary, the required sample size for this trial with two placebo periods was calculated to be 30 volunteers, with 90% overall power maintained if up to three volunteers discontinued the trial prematurely. The calculation was based on considerations for a corresponding TQT trial with only one placebo period, which would achieve the same power with 36 completing subjects, using the following assumptions:

1) An expected difference of approximately 2 ms in the primary endpoint between empagliflozin and placebo, and its common standard deviation of 14 ms.

2) The power of about 95% for testing the primary endpoint in each dose of empagliflozin against placebo to achieve an overall power of 90% for the trial, as the null hypotheses of both primary tests were to be rejected simultaneously.

The resulting sample size was also sufficient to detect a treatment difference between moxifloxacin and placebo of 8 ms in the mean QTcN change from baseline, with a power of about 95%. Moreover, the sample size has also been demonstrated to provide sufficient power to investigate the secondary endpoints (changes in QTcN interval over time) in previous trials [33]–[35].

### Randomisation

A specific Williams design was selected (see Figure 5 in [22]), which ensured that no volunteer would receive both placebo treatments in either the first two or the last two of the five periods. The assignment of the four study treatments to the five symbols (A–E) and the generation of the randomisation schedule were performed at an independent site to ensure treatment administration was double-blind (except for moxifloxacin, which was administered open-label). Volunteers were assigned to three cohorts with 10 volunteers each in agreement with the randomisation blocking factor of 10.

The randomisation list was generated using a validated software system (Clinical Trial Supply System Propack Data CTM, Version 3.3), and the resulting allocation of treatment sequences to study subjects was both reproducible and non-predictable. Access to the randomisation code was restricted until the trial was completed and the database was locked.

### Statistical analyses

Adaptations of the conventional analysis in TQT studies were implemented to account for the inclusion of two placebo periods. Statistical analyses were planned as described previously [22].

Briefly, the primary analysis compared changes in the primary endpoint (QTcN MCfB over 1–4 hours) using an analysis of covariance (ANCOVA) model with ‘sequence’, ‘period’ and ‘treatment’ as fixed effects, ‘subjects nested within sequences’ as a random effect, and ‘pre-dose baseline’ as a covariate. Analyses were performed using pair-wise comparisons of the trial treatments [36,37]. The saturated repeated measurements crossover (RMC) model with unstructured covariance using pair-wise comparisons of the trial treatments was used to evaluate changes from baseline in QTcN at any time point between 0.5–24 hours after dosing [36]. In this trial, the placebo corrected MCfB was determined using the data from both placebo periods and the test treatment simultaneously in the ANCOVA model. For descriptive statistics and the exposure-response analysis, the changes from baseline in QTcN of the placebo periods were averaged prior to the derivation of the placebo corrected MCfB.

In agreement with ICH E14, all tests were performed one-sided against the threshold level of regulatory concern of 10 ms with type-I error of α=5%, which is statistically equivalent to the calculation of two-sided 90% confidence intervals for the adjusted mean estimates. As the null hypothesis was tested simultaneously for both dosage regimens, no alpha adjustment was required taking into account the partition principle. In addition, an exposure-response analysis between empagliflozin concentrations and placebo-corrected QTcN change from baseline was carried out [38].

Sensitivity analyses included the adjustment for global average baseline [39], and direct analysis of the QT interval with RR interval as additional covariate within the ANCOVA analysis (one-step procedure [34,36,39,40]). Subgroup analyses included an analysis of the primary and secondary endpoints with respect to gender [41]. Assay sensitivity was tested using the placebo-corrected MCfB in QTcN 2–4 hours post-dose (global test [32]) for moxifloxacin 400 mg using a one-sided superiority test. No multiplicity adjustments were necessary for the primary and secondary analyses (intersection–union test [42]).

Categorical endpoints such as AEs, cardiologic assessments and occurrence of ECG intervals beyond thresholds of regulatory concern (e.g. QTc >450 ms) were analysed based on incidence rates adjusted for the number of periods in which the treatment was given. Additionally, the analysis of such events in periods with any of the two doses of empagliflozin compared with the two placebo periods provides a direct comparison of incidence rates [22]. All analyses were performed on the “full analysis set”, which comprised all recorded data of all subjects who received at least one dose of study treatment and had at least one ECG endpoint assessment at baseline and post treatment. No imputation for missing data was planned or performed.

## Results

### Patient characteristics

**Figure 1:**
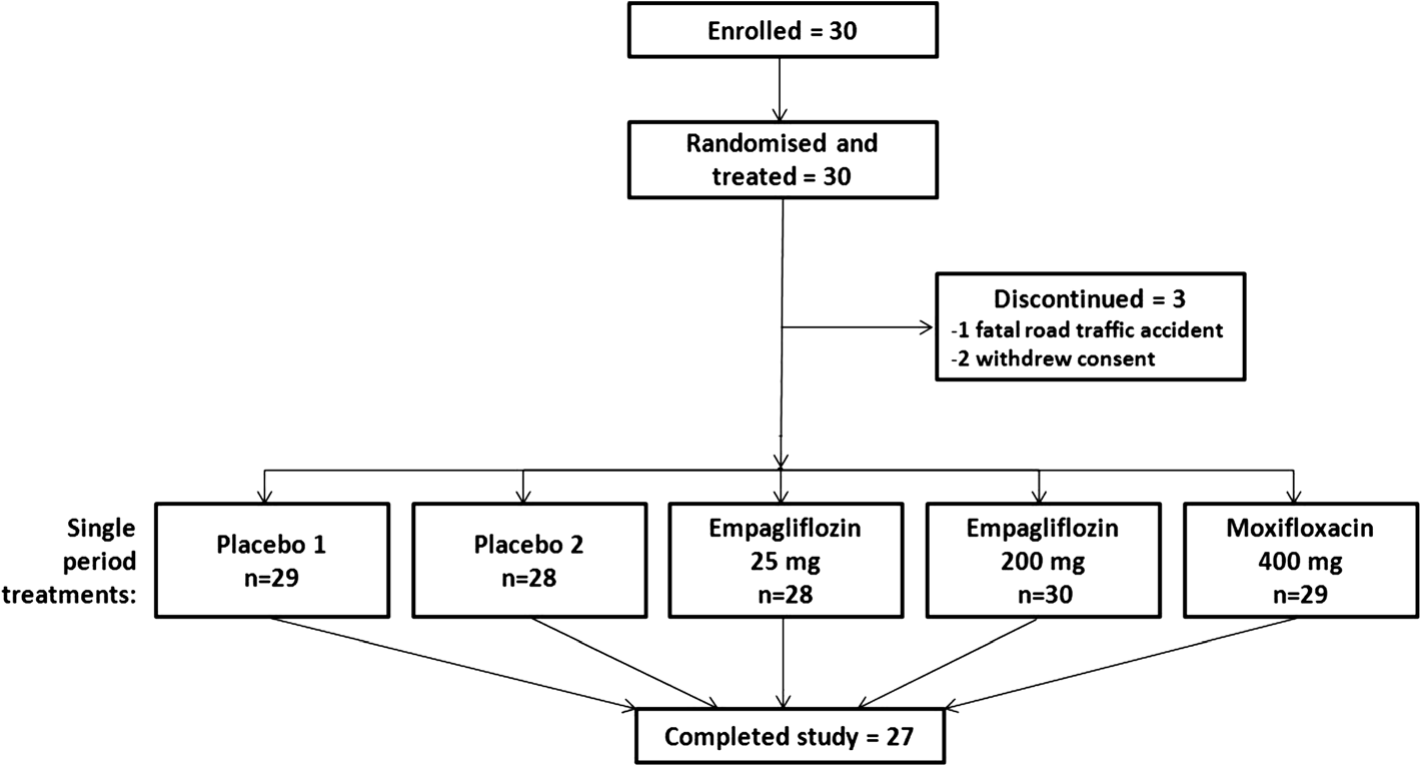
Patient flow (five-period crossover design).

Thirty Caucasian volunteers (16 male [53.3%] and 14 female [46.7%]) were included in the study; demographics and baseline characteristics are presented in Table 2. Twenty-seven volunteers completed the study; one female volunteer died in a car accident four days after administration of moxifloxacin in treatment period four, while two male volunteers withdrew consent during the study (Figure 1).

**Table 2:** Demographics and baseline characteristics (treated set)

**Randomised volunteers, n**	**30**
Male gender, n (%)	16 (53.3)
Number completing study, n (%)	27 (90.0)
**Baseline characteristics**	**Median (range)**
Age (years)	32.5 (18−52)
Height (cm)	170.5 (161−184)
Weight (kg)	68.0 (52−88)
BMI (kg/m^2^)	23.2 (19.1−28.4)
Heart rate (bpm)	56.0 (42.0−75.0)
QT interval (ms)	403.5 (363.0−475.0)
QTcN interval (ms)	399.0 (372.5−440.3)

*BMI*, body mass index; *bpm*, beats per minute; *QTcN*, population heart rate-corrected QT interval.

### Primary endpoint

The placebo-corrected MCfB in QTcN 1–4 hours after dosing were 0.6 (90% confidence interval [CI]: -0.7, 1.9) ms for 25 mg empagliflozin and -0.2 (-1.4, 0.9) ms for 200 mg empagliflozin (Table 3). Hence, the upper limit of both 90% CIs was below the pre-defined (ICH E14) threshold of 10 ms, indicating no clinically relevant prolongation in the mean QTcN interval 1–4 hours after administration of either 25 mg or 200 mg empagliflozin, compared with placebo.

**Table 3:** Adjusted mean (90% CI) values for QTcN and heart rate mean changes from baseline

		**QTcN mean change from baseline: ΔQTcN (ms)**	**Difference from placebo: ΔΔQTcN (ms)**		**HR mean change from baseline: ΔHR (bpm)**	**Difference from placebo: ΔΔHR (bpm)**	
**Treatment**	**n**	**Adjusted mean (SE)**	**Adjusted mean (SE)**	**90% CI (lower, upper)**	**Adjusted mean (SE)**	**Adjusted mean (SE)**	**90% CI (lower, upper)**
**Placebo**	29	3.7 (1.0)	0.6 (0.8)	(-0.7, 1.9)	-0.3 (0.4)	-0.9 (0.5)	(-1.8, 0.0)
**Empagliflozin 25 mg**	28	4.3 (1.1)			-1.2 (0.5)		
**Placebo**	29	3.7 (0.9)	-0.2 (0.7)	(-1.4, 0.9)	-0.3 (0.4)	0.0 (0.5)	(-0.9, 0.8)
**Empagliflozin 200 mg**	30	3.4 (0.9)			-0.3 (0.5)		
**Placebo**	29	3.5 (1.1)	12.4 (1.0)	(10.7, 14.1)	-0.2 (0.4)	2.2 (0.6)	(1.1, 3.3)
**Moxifloxacin 400 mg**	29	16.0 (1.2)			2.0 (0.6)		

Adjusted means and upper and lower 90% confidence intervals (CI) for the mean population heart rate-corrected QT interval (QTcN) changes from baseline and for the mean heart rate (HR) changes from baseline between 1 and 4 hours after administration of 25 mg or 200 mg doses of empagliflozin or placebo and between 2–4 hours after administration of 400 mg moxifloxacin or placebo. Data from the full analysis set. SE, standard error; bpm, beats per minute.

### Secondary endpoints

**Figure 2:**
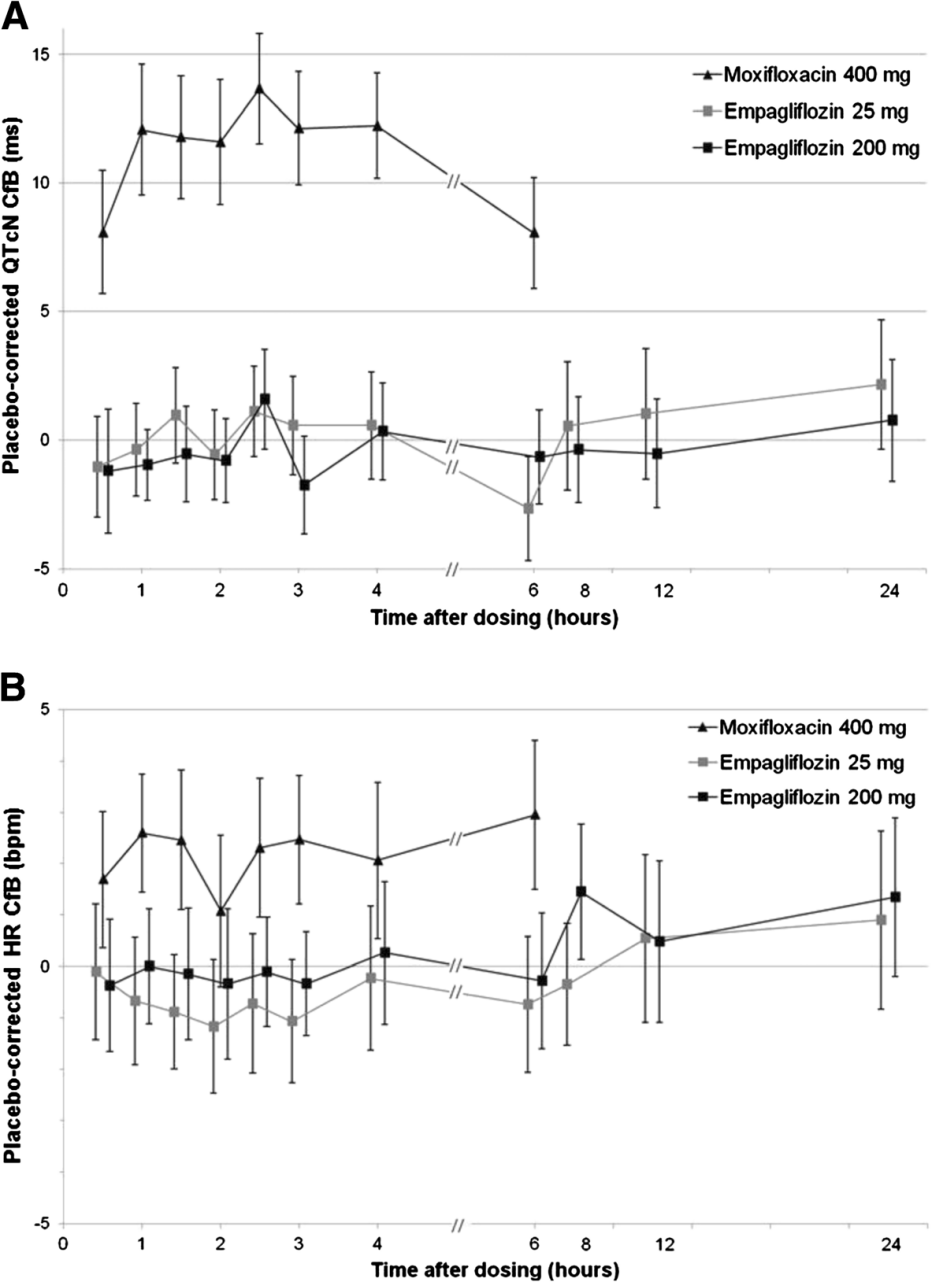
**Placebo-corrected QTcN CfB (A) and placebo-corrected heart rate CfB (B).** Data are adjusted means and 90% confidence intervals (CIs) after administration of empagliflozin 25 mg, or 200 mg, or moxifloxacin 400 mg. Data from the full analysis set analysed: placebo (n=29); 25 mg empagliflozin group (n=28); 200 mg empagliflozin group (n=30); and 400 mg moxifloxacin group (n=29). CfB, change from baseline; HR, heart rate; QTcN, population heart rate-corrected QT interval.

The placebo-corrected MCfB in QTcN from all ECGs taken between 0.5–24 hours after dosing was 0.7 (90% CI: -0.4, 1.7) ms for 25 mg empagliflozin and -0.2 (-1.2, 0.9) ms for 200 mg empagliflozin. The time courses of the adjusted means of the placebo-corrected QTcN changes from baseline for each active treatment are presented in Figure 2A. Compared with placebo, the adjusted mean values ranged from -2.7 to 2.2 ms after dosing with 25 mg empagliflozin and -1.8 to 1.6 ms with 200 mg empagliflozin. Maximum upper CIs were 4.7 and 3.5 ms, respectively, clearly below the pre-defined threshold of 10 ms.

Assay sensitivity was confirmed by placebo-corrected MCfB in QTcN 2–4 hours post-dose of 12.4 (10.7, 14.1) ms with moxifloxacin 400 mg (Figure 2A, Table 3).

### Sensitivity analyses

Based on regulatory requirements, a number of sensitivity analyses were carried out. Additional analyses included an ANCOVA model with a global baseline, recommended by Kenward and Roger [39], and a one stage-analysis of QT prolongation [40] or the Patterson model [43]. Results of these analyses were in agreement with the primary and secondary analysis of the trial.

### Other ECG parameters

Additional analyses were performed for heart rate and other heart rate-corrected QTc endpoints (QTcI, individual heart rate-corrected QT interval; QTcF, Fridericia’s correction formula; QTcB, Bazett’s correction formula). The estimated parabolic slope for the study population correction method (QTcN) was 0.294, and thus slightly lower than the slope used with the Fridericia method (0.333). The estimated slopes for the individual correction method ranged from 0.174 to 0.421.

For both empagliflozin doses, the time courses for the adjusted means of the placebo-corrected heart rate change from baseline ranged from -1.2 to 1.5 bpm, and all 90% CIs were between -3 to 3 bpm (Figure 2B). As the changes in heart rate were small (Table 3), the results of the uncorrected QT interval and other heart rate-corrected QT intervals were very similar to those of the primary and secondary analyses.

In the categorical analysis on QTc endpoints, five volunteers exceeded the QTcN threshold of 450 ms during the treatment period (0.5–24 hours post-dose for empagliflozin, and 0.5–6 hours post-dose for moxifloxacin); with one (3.6%) volunteer taking 25 mg empagliflozin, two (6.7%) volunteers taking 200 mg empagliflozin and two (6.9%) volunteers taking moxifloxacin. QTcN did not exceed 450 ms in any volunteers taking placebo (averaged data from both placebo periods), and QTcN did not exceed 480 ms in any volunteer. Five volunteers had a change in QTcN more than 30 ms, all of whom were taking moxifloxacin.

Individual ECG data were analysed for notable changes from the pre-dose assessments in heart rate, PR, and QRS intervals. These were defined as heart rate percentage change ≥25% and observed heart rate value <50 bpm or >100 bpm; PR percentage increase ≥25% and observed PR value >200 ms; QRS percentage increase ≥10% and observed QRS value >110 ms. No individual presented a notable change in any of these categories, and there were no clinically relevant findings in placebo-adjusted changes from baseline.

### Gender effects

Analysis of the primary and secondary endpoints by gender also demonstrated the absence of a clinically relevant placebo-corrected change from baseline in QTcN after administration of 25 mg and 200 mg doses of empagliflozin. Despite the low sample sizes in each gender subgroup, all 90% CIs of the primary and secondary endpoints were between -7 and 7 ms in each of the subgroups, and no clinically relevant differences between male and female volunteers were noted.

### Pharmacokinetic parameters

Following oral administration, empagliflozin was rapidly absorbed, reaching median peak levels at approximately 1.5 and 1.8 hours with the 25 mg and 200 mg doses, respectively (Table 4). Thus, for both doses, C_max_ was reached within the pre-defined three-hour time window for ECG measurements for the primary endpoint. Empagliflozin exposure increased approximately dose proportionally with the two tested doses (Table 4).

**Table 4:** Pharmacokinetic parameters for single empagliflozin doses of 25 mg and 200 mg

**Parameter**	**Empagliflozin 25 mg**		**Empagliflozin 200 mg**	
	**gMean**	**% gCV**	**gMean**	**% gCV**
**AUC**_**0−tz**_**(nmol∙hour/L)**	4860	16.7	36400	20.0
**C**_**max**_**(nmol/L)**	768	23.2	4860	22.1
	**Median**	**Range**	**Median**	**Range**
**t**_**max**_**(hours)**	1.5	0.5–4.0	1.8	1.0–4.1

AUC_0–tz_, area under the concentration-time curve of empagliflozin in plasma over the time interval 0–time of the last measurable concentration of empagliflozin in plasma; C_max_, maximum plasma concentration of empagliflozin; and t_max_, time to maximum plasma concentration of empagliflozin. Data from the full analysis set analysed per protocol: 25 mg empagliflozin dose group (n=28), 200 mg empagliflozin dose group (n=30). gMean, geometric mean; gCV, geometric coefficient of variance.

### Pharmacokinetic-pharmacodynamic evaluation

**Figure 3:**
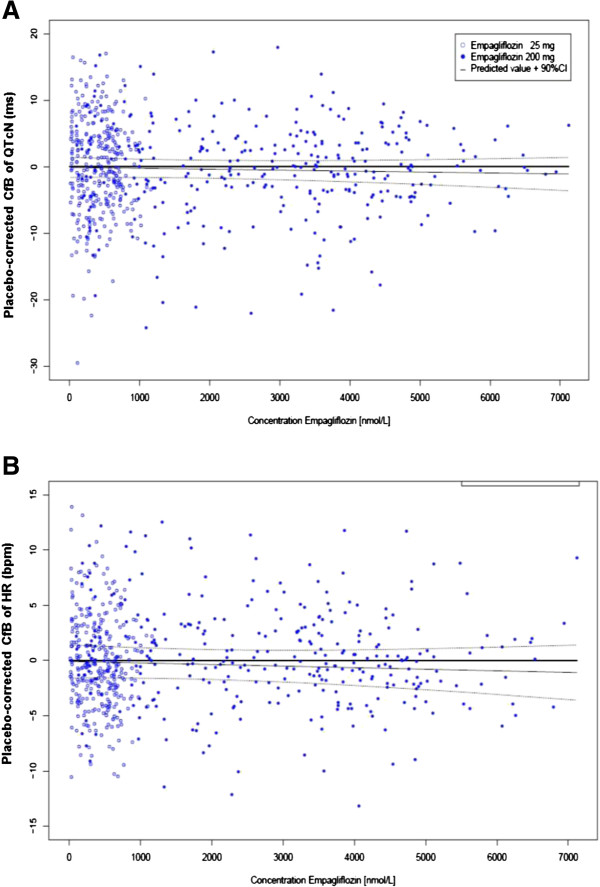
**Empagliflozin exposure-response relationships for placebo-corrected QTcN (A) and heart rate (B) changes from baseline.** Placebo-corrected changes from baseline versus plasma concentrations of empagliflozin for empagliflozin 25 mg and empagliflozin 200 mg treatment groups. HR, heart rate; QTcN, population heart rate-corrected QT interval. Data from the full analysis set.

The exposure-response analysis for placebo-corrected QTcN change from baseline for both empagliflozin doses resulted in slope estimates that were zero or close to zero and their two-sided 95% CIs included zero (Table 5; Figure 3A). Similar results were obtained for the relationship between empagliflozin and heart rate, with slope estimates close to zero and CIs that include zero for both doses (Figure 3B). These results demonstrate no relationship between empagliflozin exposure and either prolongation of the QTcN interval or change in heart rate compared with placebo.

**Table 5:** Slope and intercept from the exposure-response analysis of placebo-corrected QTcN change from baseline by dose

		**Slope [ms/(nmol/L)]**			**Predicted value of placebo-corrected change from baseline QTcN (ms) at gMean of C**_**max**_	
**Treatment**	**Intercept (ms)**	**Estimate**	**95% CI**	**gMean of C**_**max **_**(nmol/L)**	**Estimate**	**90% CI**
**Empagliflozin 25 mg**	0.35	-0.0007	-0.0043, 0.0029	768	-0.20	-1.65, 1.24
**Empagliflozin 200 mg**	-0.60	-0.0000	-0.0005, 0.0005	4860	-0.64	-2.33, 1.05

Predicted values and confidence intervals (CI) determined by the exposure-response relationship of empagliflozin (25 mg or 200 mg treatment group). Data from the full analysis set. QTcN, population heart rate-corrected QT interval.

### Safety and tolerability

Overall, 15 of 30 volunteers (50%) experienced AEs during the trial; three of 28 volunteers (10.7%) taking 25 mg empagliflozin, five of 30 (16.7%) taking 200 mg empagliflozin, eight of 29 (27.6%) taking placebo, and one of 29 (3.4%) taking moxifloxacin. None were considered by the investigator to be related to the study medication.

The one serious AE leading to study discontinuation was the fatal car accident in one participant receiving moxifloxacin. In addition, two other volunteers developed severe AEs (nasopharyngitis of severe intensity on placebo and headache of severe intensity on 25 mg empagliflozin).

The most frequent AE was nasopharyngitis, reported by nine volunteers: five (17.2%) taking placebo, two (7.1%) taking 25 mg empagliflozin, and two (6.7%) taking 200 mg empagliflozin. Other AEs included headache in one volunteer taking 25 mg empagliflozin (3.6%) and two taking placebo (6.9%) and oropharyngeal pain in one volunteer taking 200 mg empagliflozin (3.3%) and one taking placebo (3.4%). Nausea, vomiting and skin rash AEs were each reported in one volunteer (3.3%) taking 200 mg empagliflozin. The remaining AEs (arthropod bite and car accident) were each experienced by one volunteer (3.4%) taking placebo and moxifloxacin, respectively.

### Efficiency of the new trial design

Another sensitivity analysis compared the effect of the pooled, double placebo design with the use of single placebo periods in the primary and secondary analyses. The results for the primary analysis are shown in Table 6. As expected [22], the standard error of the placebo-corrected change from baseline of QTcN was inflated by about 15%, on average, when only one of the placebo periods was used in the primary and secondary analyses. This demonstrated that the assumptions for using this design [22] were fulfilled; i.e. that the changes from baseline showed low intra-individual correlation between the two placebo periods.

**Table 6:** Additional sensitivity analysis comparing use of 1 or 2 placebo periods for the primary analysis

**Placebo-corrected adjusted means and 90% CIs for the mean QTcN change from baseline 1−4 hours after dosing analysed using two different ANCOVA models**				
**ANCOVA model**	**25 mg empagliflozin Difference from placebo, ms**		**200 mg empagliflozin Difference from placebo, ms**	
	**Mean (SE)**	**90% CI**	**Mean (SE)**	**90% CI**
Primary analysis model with placebo period 1 only	0.2 (0.9)	(-1.4, 1.8)	-0.5 (0.8)	(-1.8, 0.8)
Primary analysis model with placebo period 2 only	0.6 (1.0)	(-1.2, 2.4)	0.1 (0.8)	(-1.4, 1.5)

Data from full analysis set (n=30). ANCOVA, analysis of covariance; CI, confidence interval; QTcN, population heart rate-corrected QT interval.

## Discussion

The prolongation of cardiac repolarisation, as measured by the QT interval, can potentially increase the probability of fatal cardiac arrhythmia [44]. As such, TQT studies of new drugs are recommended by regulatory guidelines in order to evaluate the potential effects of new drugs on cardiac repolarisation [12,24]. TQT studies determine whether the drug has a threshold pharmacologic effect on cardiac repolarisation, as detected by QT/QTc prolongation [12,24]. A negative TQT study is indicated when the upper bound of the 95% one-sided CI for the largest time-matched mean effect of the drug on the QTc interval excludes 10 ms. TQT studies are typically carried out in healthy subjects at early stages of drug development [12,24]. Further investigation in the target patient population is generally only required following a positive TQT study; following a negative TQT study, the collection of baseline and periodic on-therapy ECGs during subsequent stages of drug development is generally sufficient [12,24]. There are some data to suggest that there may be inter-ethnic differences in drug-induced QT-prolongation effects [45], and that risk of QT interval prolongation may be increased in females, patients with organic heart diseases and patients with hypokalaemia [46], but further investigation of these factors is required.

This randomised, double-blind, placebo-controlled trial (with open-label moxifloxacin), employing a new five-period crossover design with two placebo periods, aimed to assess the effects of empagliflozin on QT interval in healthy volunteers, according to the ICH E14 guideline [12,24]. Empagliflozin was found to have no effect on population heart rate-corrected QTc interval length measured in healthy volunteers at either therapeutic (25 mg) or supratherapeutic (200 mg) doses. The placebo-corrected QTcN interval changes from baseline after administration of 25 mg or 200 mg empagliflozin were below the threshold of regulatory concern of 10 ms, both in the pre-defined interval, as well as over the whole time course. In addition, there were no clinically relevant findings regarding the placebo-corrected changes from baseline for heart rate or any of the other heart rate-corrected QTc intervals that were investigated. Subgroup analyses by gender also demonstrated the absence of a significant effect of treatment on the primary endpoint (mean change from baseline in the QTcN between 1–4 hours after dosing). Furthermore, exposure-response analysis confirmed the lack of a relationship between empagliflozin exposure and QT interval length. No other clinically relevant changes in ECG parameters were observed after empagliflozin administration. Also assay sensitivity was shown with QTc effects of moxifloxacin, which were consistent with previous reports [25].

The five-period crossover design employed in this TQT study has been shown to be more efficient than the usual four-period design for TQT trials. The design is based on the objective that all comparisons are performed between active drugs and placebo, and no comparisons between active drug groups are necessary [22]. Furthermore, the use of two placebo periods increases the number of measurements taken while on placebo, thus reducing the variability of placebo estimates and of placebo-corrected values for active treatment groups. As a consequence, a 25% smaller sample size is required to achieve the same power as the corresponding four-period trial design [22,23,47].

The sample size required to maintain an overall power of 90% for this five-period TQT trial was 30 subjects (including three potential drop-outs), compared with 40 subjects (including four potential drop-outs) in a conventional four-period design [22]. Moreover, the number of ECG recording sessions was reduced by 7% (150 compared with 160), leading to a reduction in cost and effort of a similar magnitude.

The results obtained from ECG recordings in this study are consistent with a lack of relevant ECG-related effects observed during the pre-clinical and clinical development of empagliflozin to date. *In vitro* and animal studies with empagliflozin demonstrated no relevant interactions with the hERG-mediated potassium current and no effect on action potentials (unpublished data). There were also no clinically relevant changes to ECG recordings made in clinical trials of both healthy volunteers [10] and patients with T2DM [48]. These clinical studies also noted an absence of any relevant placebo-corrected changes from baseline in heart rate after empagliflozin administration. The cardiovascular safety of empagliflozin in patients with T2DM continues to be studied as part of the Phase III study program and is being investigated in a dedicated cardiovascular outcome trial (NCT01131676).

The results from the pharmacokinetic analysis of empagliflozin in the current study are consistent with the findings of previous studies in healthy volunteers [10] and patients with T2DM [48,49]. The safety findings of this study were also in line with previous clinical studies conducted in both healthy volunteers and patients with T2DM [8,10,48]. Single doses of empagliflozin were well tolerated. The majority of AEs were mild to moderate in severity (the most frequent being nasopharyngitis) and none were considered to be related to study medication.

## Conclusions

In conclusion, this study, conducted according to ICH E14 guidance, has shown that empagliflozin was not associated with QTc interval prolongation at therapeutic and supratherapeutic doses, and was well tolerated by male and female healthy volunteers. The new double-placebo period study design proved to be efficient for TQT trials.

## Abbreviations

AE: Adverse event; ANCOVA: Analysis of covariance; AUC0−tz [g·h/mL]: Area under the concentration-time curve of the analyte in plasma over the time interval 0 to tz; BMI [kg/m2]: Body mass index (weight divided by height squared); CI: Confidence interval; Cmax [g/mL]: Maximum measured concentration of the analyte in plasma; CV [%]: Coefficient of variance; CVD: Cardiovascular disease; ECG: Electrocardiogram; HEK293 cells: Human embryonic kidney cells; hERG: Human ether-a-go-go related gene; HPLC-MS/MS: High performance liquid chromatography, tandem mass spectrometry; HR [bpm]: Heart rate; MCfB: Mean change from baseline; PR [ms]: Interval between the onset of the P wave and the start of the QRS complex, representing the time the impulse takes to reach the ventricles from the sinus node; QRS [ms]: Interval between the onset of the Q wave and the end of the S wave, representing the duration of ventricular depolarisation; QT [ms]: The interval between the onset of the Q wave and the end of the T wave, representing the duration from the depolarisation to the repolarisation of the ventricles; QTc [ms]: Corrected QT interval; QTcB [ms]: QT interval corrected for heart rate using Bazett’s correction formula; QTcF [ms]: QT interval corrected for heart rate using Fridericia’s correction formula; QTcI [ms]: Individual heart rate-corrected QT interval; QTcN [ms]: Population heart rate-corrected QT interval; RMC: Repeated measurements crossover model; RR [ms]: Interval between subsequent R waves; SE: Standard error; SGLT2: Sodium glucose cotransporter 2; T2DM: Type 2 diabetes mellitus; tmax [h]: Time from (last) dosing to C_max_; TQT: Thorough QT study.

## Competing interests

AR, TB, SM, GS, HJW and UCB were employees of Boehringer Ingelheim at the time of conduct and reporting of the study; BW and KBG were contracted by Boehringer Ingelheim for analysis and reporting.

## Authors’ contribution

The authors meet criteria for authorship as recommended by the International Committee of Medical Journal Editors (ICMJE), were fully responsible for all content and editorial decisions, were involved at all stages of manuscript development, and have approved the final version. AR, TB, SM, HJW and UCB designed the study. AR, KBG, GS, BW, TB and SM released the statistical analysis plan. AR, GS and BW were responsible for the statistical analyses. All authors participated in the preparation of the clinical trial report. All authors read and approved the final manuscript.
